# Supplementary material for: Characterisation of the Gastrointestinal Microbiome of Green Sea Turtles (Chelonia mydas): A Systematic Review
Source: Animals (Basel). 2025 May 29;15(11):1594. doi: 10.3390/ani15111594 (PMC12153662; doi:10.3390/ani15111594)
Supplement: Supplementary file 1 [file animals-15-01594-s001.zip › animals-3552361-supplementary.pdf]

Supplementary Materials

Characterisation of the Gastrointestinal Microbiome of Green Sea Turtles (*Chelonia mydas*): A Systematic Review

Dawood Ghafoor [1], Orachun Hayakijkosol [1,2], Carla Ewels [3] and Robert Kinobe [1,2,4,*]

1 Veterinary Preclinical Sciences, College of Science and Engineering, James Cook University, Townsville, QLD 4811, Australia; dawood.ghafoor@my.jcu.edu.au (D.G.); orachun.hayakijkosol1@jcu.edu.au (O.H.)
2 Centre for Tropical Biosecurity, James Cook University, Townsville, QLD 4811, Australia
3 Statistics and Data Sciences, College of Science and Engineering, James Cook University, Townsville, QLD 4811, Australia; carla.ewels@jcu.edu.au
4 Centre for Molecular Therapeutics, Australian Institute of Tropical Health and Medicine, James Cook University, Townsville, QLD 4811, Australia
* Correspondence: robert.kinobe@jcu.edu.au; Tel.: +61-7-47814061

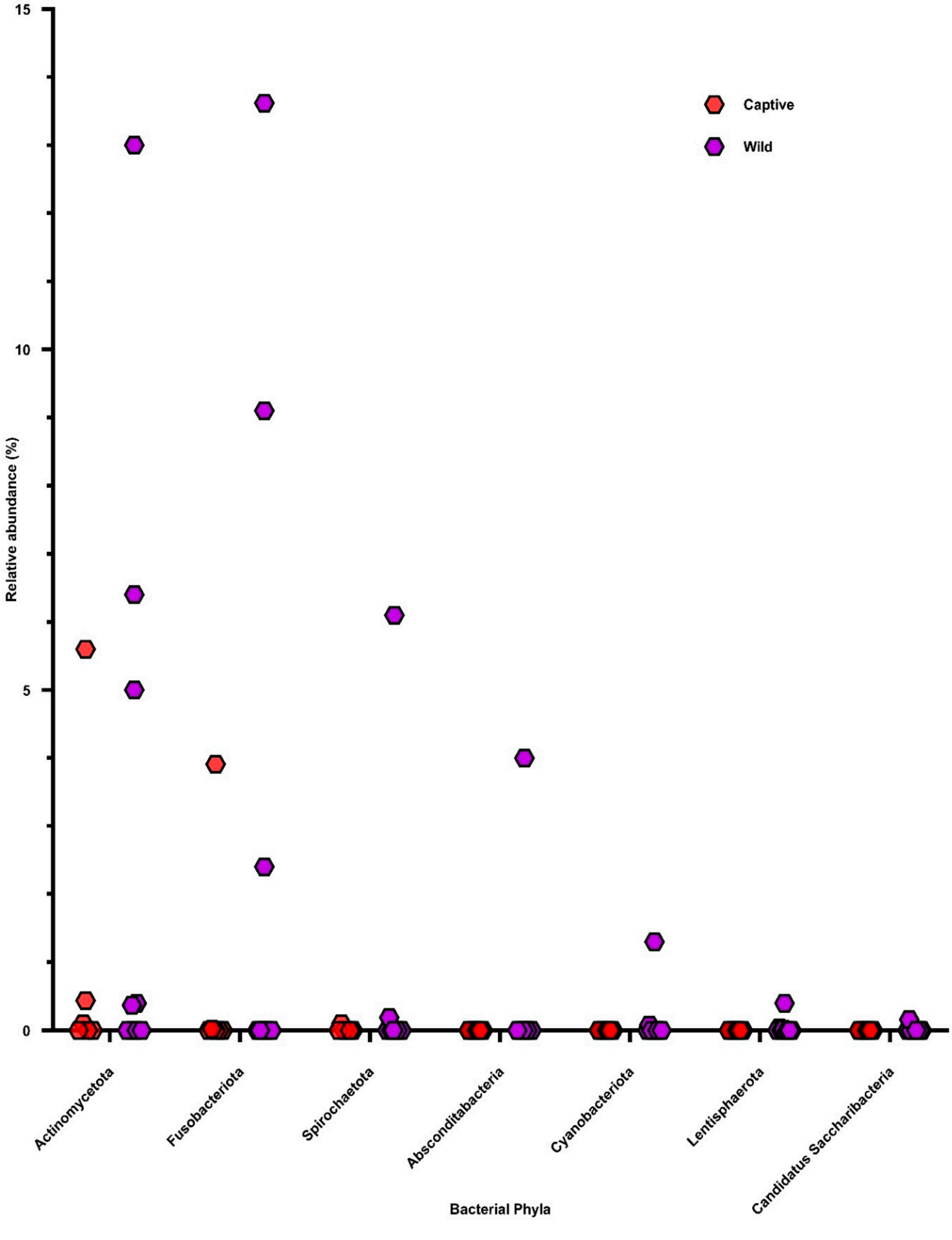

Figure S1: Relative abundance of less dominant bacterial phyla across studies included in the systematic review.

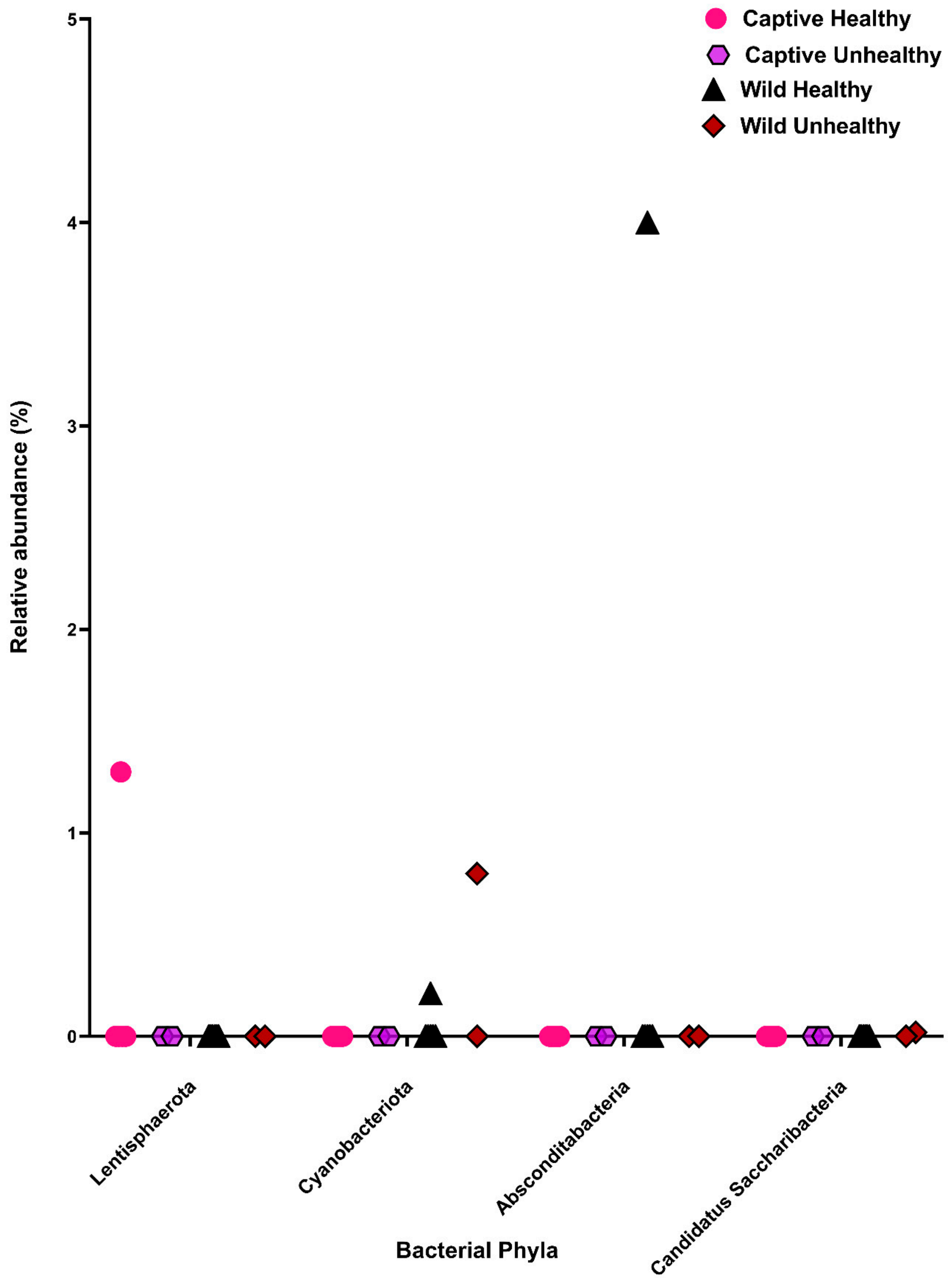

Figure S2: Distribution of phyla with very low relative abundance in captive and wild green sea turtles under different health conditions.

Table S1. The table outlines the studies that met the inclusion criteria for data extraction and evaluation against each essential qualitative measure. The rightmost column numbers show the number of studies that met each qualitative criterion, whereas the bottom row percentages exhibit the cumulative qualitative score for each study.

No.	Feature	[11]	[12]	[17]	[19]	[20]	[21]	[31]	[32]	[33]	[34]	[35]	[36]	[37]	
1	Well-defined study rationale and objectives	✓	✓	✓	✓	✓	✓	✓	✓	✓	✓	✓	✓	✓	13/13
2	Geographical location of study explicitly mentioned	✓	✓	✓	✓	✓	✓	✓	✓	✓	✓	✓	✓	✓	13/13
3	Defined sampling strategy and sample size	✓	✓	✓	✓	✓	✓	✓	✓	✓	✓	✓	✓	✓	13/13
4	Age group or life stage of turtles clearly stated	✓	x	✓	✓	x	x	✓	✓	✓	✓	✓	x	✓	9
5	Clearly stated whether the turtles are wild or captive	✓	✓	✓	✓	✓	✓	✓	✓	✓	✓	✓	✓	✓	13/13
6	Health status of turtles explicitly defined	✓	✓	✓	✓	✓	✓	✓	✓	✓	✓	✓	✓	✓	13/13
7	Taxonomic classification provided at the phylum level	✓	✓	✓	✓	✓	✓	✓	✓	✓	✓	✓	✓	✓	13/13
8	Classifications at genus, class, or both levels	✓	x	✓	✓	x	✓	✓	x	✓	✓	✓	✓	✓	10/13
9	Microbiome analysis method clearly described	✓	✓	✓	✓	✓	✓	✓	✓	✓	✓	✓	✓	✓	13/13
10	Gut microbiome diversity qualitatively described	✓	✓	✓	✓	✓	✓	✓	✓	✓	✓	✓	✓	✓	13/13
	Overall Qualitative Measure (%)	100	80	90	100	80	90	100	90	100	100	100	100	100	

Table S2: Summary of the sampling timeline, life cycle stage, population type, study interventions, rehabilitation status, number of samples, and corresponding references. N/A indicates data not available

No	Sampling Timeline	Sea Turtle Life Stages / Curved Carapace Length (CCL)	Population Type	Study Interventions	Rehabilitation Status	Diet	Sample Size	References
1	N/A	Juvenile and Sub-adult	Wild	Comparison of bacterial communities between pre-hospitalization (PH) and post-rehabilitation (PH) stranded turtles.	Healthy	Human grade seafood such as squid	4	[11]
2	October 2019	CCL Range 36.5 to 48.1 cm	Wild	Analysed microbial communities on algae and seagrass, evaluating their role in turtle digestive microbiomes and establishing baselines for GI compartments and food items.	Healthy and Dead	N/A	7	[12]
3	N/A	Juvenile and Adult	Wild	Characterised and compared mucosa-associated bacterial communities in various regions of the gastrointestinal tract of stranded green turtles.	Stranded and died 1-4 days after arrival	Human grade seafood such as squid	4	[17]
4	January 2014 -	Juveniles	Wild	Characterised genotypic bacterial	Healthy		17	[19]

	January 2016			community composition in feces from juvenile green turtles over time, focusing on changes related to variations in diet during rehabilitation.				
5	February to March 2016	CCL Range 31.1 to 64.7 cm	Wild and Captive	Investigated microbiota in feces and rectum of wild and captive green turtles from Brazil, focusing on dietary shifts, regional differences, and omnivorous diets.	5 Dead and 19 Healthy	Macroalgae and fish	24	[20]
6	September 2015 (wild) October 2015 and March 2016 (stranded)	CCL Range 41.3–87.6	Wild and Captive	Compared fecal bacteria in wild turtles from Bowen/Townsville with those in stranded turtles to understand potential health implications.	Stranded (wild) and Healthy (captive)	N/A	12	[21]
7	N/A	Juvenile	Captive	Evaluated bacteriophage therapy versus enrofloxacin antibiotics for targeting Acinetobacter in green turtles and assessed its impact on gut bacterial diversity.	Healthy	Nutrient-enriched seafood-vegetable diet	12	[31]

8	January 2017	Adult Female Turtles (Nesting)	Wild	Categorised microbiota of sea turtle species, explored relationships between turtle phylogeny and microbiota composition, and examined the evolution of microbiotas.	Healthy	N/A	18	[32]
9	July 13-18, 2015 (pelagic); Sept-Oct 2015 (neritic)	Juvenile	Wild	Characterised the cloacal microbiome of green turtles in the context of their ontogenetic shifts.	Healthy	N/A	9	[33]
10	Late Fall/Early Winter 2019	Juvenile	Wild	Compared fecal and cloacal bacterial communities of cold-stunned turtles from three species, hypothesising differences between species and gut locations.	Cold-stunned stranded	N/A	5	[34]
11	July 2020	CCL Range 60.5 to 79 cm	Captive	Compared gut microbiota of hawksbill and green sea turtles undergoing rehabilitation.	Healthy	Fish and Plants based food	9	[35]
12	July 2020	CCL Range 52 to 60.5 cm	Captive	Compared gut microbiota of hawksbill and green	Healthy	Fish and Plants based food	3	[36]

				sea turtles undergoing rehabilitation.				
13	Between March and April 2022	Juvenile	Captive	Identified the causative agent of ulcerative carapacial disease in juvenile green turtles by analysing microbial diversity in carapacial ulcers, healthy carapaces, feces, and water.	Healthy	Vegetables (lettuce or cabbage), and squid	15	[37]
